# Surface Modification of TiO_2_ and ZrO_2_ Nanoparticles with Organic Acids and Ultrasound to Enhance Antibacterial Activity

**DOI:** 10.3390/ma18122786

**Published:** 2025-06-13

**Authors:** Guadalupe Tellez-Barrios, Gregorio Cadenas-Pliego, Iván Toledo-Manuel, Marissa Pérez-Alvarez, Carmen N. Alvarado-Canche, Sergio Mancillas-Salas, Marlene Andrade-Guel, José Manuel Mata-Padilla, Christian Javier Cabello-Alvarado

**Affiliations:** 1 Centro de Investigación en Química Aplicada (CIQA), Blvd. Ing. Enrique Reyna H. No. 140, Saltillo Coahuila C.P. 25294, Mexico; guadalupe.tellez.d24@ciqa.edu.mx (G.T.-B.); ivan.toledo.d22@ciqa.edu.mx (I.T.-M.); carmen.alvarado@ciqa.edu.mx (C.N.A.-C.); marlene.andrade@ciqa.edu.mx (M.A.-G.); jose.mata@ciqa.edu.mx (J.M.M.-P.); christian.cabello@ciqa.edu.mx (C.J.C.-A.); 2 Universidad Politécnica de Ramos Arizpe (UPRA), Blvd. Sigma s/n, Corredor Industrial Santa María, Ramos Arizpe, Coahuila C.P. 25900, Mexico; sergio.mancillas@upra.mx

**Keywords:** metal oxide nanoparticles, surface modification, ultrasound, antimicrobial activity

## Abstract

Metal oxide nanoparticles (NPs) are known to have biological activity against various microorganisms; thus, they have been widely used as microbicidal agents, and their use poses potential solutions to problems such as biofouling. This study focuses on the surface modification of TiO_2_ and ZrO_2_ nanoparticles with lactic acid (LA) and stearic acid (SA) to enhance their antibacterial activity (AA). The surface modification of TiO_2_ and ZrO_2_ nanoparticles was performed using continuous frequency ultrasound. Sonication was performed at different reaction times. Characterization of the modified nanoparticles by TGA, DSC, XRD, FTIR, and XPS techniques demonstrated the presence of the organic ligand on the surface of the nanoparticles. The surface modification results in a reduction in the crystal size of the nanoparticles. Regarding the antibacterial properties of modified TiO_2_ and ZrO_2_ nanoparticles, their minimum bactericidal concentration (MBC) against Gram-negative and Gram-positive bacteria of the bacterial strains *Escherichia coli* and *Staphylococcus aureus* was evaluated. The results obtained from the AA of the modified and unmodified nanoparticles demonstrated greater efficacy of the modified nanoparticles, in the particular case of TiO_2_ and TiO_2_-LA, evaluated at concentrations of 200, 500, 800, 1100, and 1400 ppm, TiO_2_-LA nanoparticles showed better results at most of the concentrations studied and a bacterial inhibition percentage of 99.0% was achieved at a concentration of 500 ppm against the *Escherichia coli* bacteria, while TiO_2_ NPs only reached 55.0%, this shows that ligands with more than one functional group play an important role in improving AA.

## 1. Introduction

Metal oxide nanoparticles obtained from metal salt precursors have been widely studied. These nanoparticles present new or improved physicochemical properties compared to macroscopic particles of the same material. Furthermore, they come in various morphologies, including spherical, triangular, cubic, and hexagonal, to mention some. Titanium dioxide (TiO_2_), zinc oxide (ZnO), and tin dioxide (SnO_2_) nanoparticles have been reported to exhibit unique properties (magnetic, optical, chemical, and mechanical), which are promising applications in several scientific areas, such as electronics, optics, catalysis, sensors, in medicine, biomedicine, agriculture, environment, etc. [1,2,3].

Due to their small size, nanoparticles are characterized by having a high surface area per unit of mass [4]. The number of atoms on the surface increases exponentially as the size decreases. The total surface area increases as the volume is divided into smaller units. As a result, a high surface energy is obtained, which makes the nanoparticles very reactive [1].

TiO_2_ nanomaterials have gained the attention of researchers due to their low production cost, high thermal and chemical stability, non-toxicity, and good photocatalytic performance. Many researchers have studied the performance of TiO_2_ nanoparticles in the anti-biofouling treatment and the hydrophobicity of polymeric membranes. When nanocomposite coatings formulated with TiO_2_ nanoparticles are exposed to sunlight, they show oxidizing and reducing properties. However, TiO_2_ nanoparticles have some drawbacks for their application as anti-biofouling nanofillers in polymeric coatings. From the perspective of antimicrobial application, TiO_2_ nanoparticles can only absorb ultraviolet light (λ ˂ 400 nm, 5% of sunlight) because the band gap of anatase and rutile TiO_2_ is 3.2 and 3.0 eV, respectively. Therefore, shifting the photocatalytic efficiency of TiO_2_ towards visible light (approximately 45% of sunlight) is an approach to increase the photocatalytic efficiency of TiO_2_ nanoparticles to improve the anti-biofouling performance of the polymer [5].

Hydrophobic ZrO_2_ nanoparticles with good chemical and mechanical stability have been widely used in polymer matrices as anti-biofouling agents. In particular, ZrO_2_ nanoparticles present good catalytic, conductive, refractory, mechanical, and high corrosion resistance properties [6]. ZrO_2_ nanoparticles present improved properties in terms of low thermal conductivity, transparency in the visible range, high refractive index, scratch resistance, mechanical resistance, improved lubrication properties, increased resistance to chemical attack, and increased resistance to oxidation and aging. Among the most important applications of ZrO_2_ are the manufacture of ceramic pigments, porcelain enamels, insulating materials, optical storage, television glasses, stereo, magnetic generators, dielectric transmitters, etc. [7].

Surface modification of nanoparticles can be understood as the integration of chemical functional groups or molecules on the surface of nanocomposites. The modification leads to a change on the surface, which promotes self-organization and compatibility between the nanoparticles and the different materials into which they could be incorporated. Nanoparticles have been modified with compounds: medicinal, polymers, organic, and inorganic functional groups. Furthermore, it has been shown that the modification protects nanoparticles against agglomeration by improving their stability and dispersion [8]. Among the main functional groups that have been used for the modification of nanoparticles are thiols, disulfides, amines, nitriles, carboxylic acids, phosphines, etc. The main aim of the modification of the nanoparticles is to coat the surface with a molecule that has the chemical functionality required for the intended use. This will depend on the various applications that the nanoparticles can be used for, such as antifouling coatings, biomedical applications, membranes for wastewater treatment, food, and dental prostheses, among others. Since the surface properties of nanoparticles are drastically altered in all cases when they are modified, and since this surface property can be used to regulate their size and self-organization during their development, the surface chemistry of nanoparticles is a crucial component in their synthesis [9].

Different metal oxide nanoparticles (e.g., SiO_2_, Fe_3_O_4_, Al_2_O_3_, CuO, TiO_2_, and Fe_2_O_3_) have been surface modified with reagents such as silanes [10], terephthalic acid [11], stearic acid [12,13], thiols [13], polyethylene glycol [14], bisamine [15] and ethylenediaminetetraacetic acid [16]. Surface modification with organic agents minimizes the surface energy, stabilizes the metal oxide nanoparticles, and makes them suitable for various applications.

Polar ligands may favor applications where nanoparticles are required to be easily dispersed in polar media such as water and polar organic solvents. Several research groups have explored using small water-soluble molecules to stabilize NPs. For example, it has been reported that using citric acid favors the dispersion of Fe_3_O_4_ in water since the aqueous solutions obtained have stayed stable for more than a month [17]. This property is important in the manufacture of antibacterial water-based paints because, by preventing the agglomeration of nanoparticles, the efficiency in eliminating pathogens increases.

The use of stearic acid in the surface modification of nanoparticles is interesting because it produces hydrophobic nanoparticles that can be easily dispersed into polymers and resins. This phenomenon indicates that SA improves the compatibility of inorganic particles in the polymer matrix and promotes their interfacial adhesion. For example, the addition of SA-modified ZnTiO_3_ particles to silicone resins enhances dispersibility in silicone resins and leads to self-cleaning coatings. The contact angle of coatings increases as the percentage of modified ZnTiO_3_ particles in the resin increases. As a result, coatings show potential anti-graffiti application due to the exceptional repellency of coated surfaces against water-based ink, oily red marker, and Paint [18].

SA has also been used to form superhydrophobic surfaces in non-anodized titanium with contact angles of 160.0° [19]. Self-cleaning coatings have a strong bacterial repulsion, which substantially reduces their adhesion. These types of technologies used to keep surfaces clean are considered environmentally friendly. Coatings for self-cleaning are used in a wide range of industries, including textile, automotive, optical, marine, aerospace, construction, and medicine [20].

The formation of nanostructures with SA also eliminates pathogens efficiently; self-assembled crystals of SA on highly ordered pyrolytic graphite create mechanobactericidal surfaces that are of great interest because they can kill bacteria without the need for antibacterial chemicals [21].

The chemical structure of SA, a fatty acid (FA), includes a carboxylic group and an aliphatic chain, which has special properties that improve AA. Although several reviews describe the antibacterial activity of fatty acids (FA), the apparent multiple mechanisms that confer antibacterial activity to FA are not well understood and need to be further reviewed [22,23,24,25]. Understanding the role of SA over AA is difficult because it is often used in conjunction with multiple elements to achieve good dispersion, including antibiotics [26], synthetic polymers [27], and biopolymers [28].

In this paper, we present the surface modification of TiO_2_ and ZrO_2_ nanoparticles with LA and SA employing ultrasound processing. The antimicrobial properties of these particles will be tested in this study against the bacteria *Escherichia coli* and *Staphylococcus aureus*.

## 2. Materials and Methods

### 2.1. Materials

The nanoparticles utilized in this study were titanium dioxide (TiO_2_) nanoparticles obtained from Sigma-Aldrich, Saint Louis, MO, USA and zirconia (ZrO_2_) nanoparticles supplied by Skispring. Distilled water was obtained from CIQA. The organic acids used in this study were stearic acid and lactic acid, which were supplied by Jalmek, San Nicolás de los Garza, N.L., Mexico. The agar used in the evaluation of the antibacterial activity was Soya Trippticase from the brand Bioxon, and the microorganisms used were *Escherichia coli* (ATCC-25922) and *Staphylococcus aureus* (ATCC-29213) provided by American Type Culture Collection (ATCC), (Manassas, VA, USA).

### 2.2. Nanoparticle Modification

The surface modification of TiO_2_ and ZrO_2_ NPs was carried out similarly to what was reported [29]. The procedure is described below: In a beaker, 100 mL of distilled water was added, and 10 mL of LA or 10 g of SA was added under magnetic stirring. This solution was heated to a constant temperature of 80 °C. Once the temperature was reached, 0.5 g of nanoparticles (TiO_2_ or ZrO_2_) were added with continuous stirring to disperse the nanoparticles in the solution. After 5 min, the mixtures obtained from the nanoparticles with the respective organic acids were subjected to ultrasound treatment with a catenoid ultrasonic probe, coupled to a homemade ultrasonic generator with an output power of 750 W, a wave amplitude of 50% and a continuous frequency of 20 KHz ± 0.1 at different reaction times (30, 45, 60, and 120 min). At the end of each reaction, the solutions were heated at a constant temperature of 80 °C for 20 min. Afterward, the solutions were filtered, and the solid was washed with 50 mL of distilled water. In addition, SA-modified nanoparticles were washed three times with 50 mL distilled water at 80 °C. The nanoparticles were placed in a vacuum oven and dried at 100 °C for 24 h.

### 2.3. Characterization

For thermogravimetric analysis (TGA), a Discovery TGA 5500 (TA instruments Inc., New Castle, PA, USA) was employed. The study consisted of approximately 10 mg of the sample from 25 to 700 °C with a heating rate of 10 °C/min in an N_2_ atmosphere, and from 600 to 700 °C, the atmosphere was replaced with oxygen to promote the combustion process.

A differential scanning calorimetry analysis (DSC) was performed using a Discovery DSC 2500 (TA instruments Inc., New Castle, PA, USA) was used for this test. This analysis was performed to determine the melting and crystallization points of unmodified and modified nanoparticles. We employed 10 mg of sample, placed it in a sample holder of aluminum, and subjected it to a first heating process from 25 to 100 °C and a second heating process from 25 to 300 °C. All analyses were performed with a 10 °C/min heating and cooling ramp in an N_2_ atmosphere at 50 mL/min flow.

The wide-angle X-ray diffraction (WAXD) technique was used for studying the crystalline structure of the unmodified and modified nanoparticles, using a Siemens D-5000 model diffractometer(Siemens, Berlin, Germany) operated at a voltage of 35 kV and a current intensity of 25 mA. The scan range on the 2θ scale was 10 to 80° with a step size of 0.02°/s. The average particle diameter (APD) for the functionalized samples was determined from the WAXD diffractograms with the Debye–Scherrer formula [30].

Fourier transform infrared spectroscopy (FTIR) analysis for unmodified and modified nanoparticles was performed on a Nicolet Magna model 550 spectrophotometer by ATR (GMI, Minneapolis, MN, USA). The samples were analyzed in a spectral range of 400 to 4000 cm^−1^.

For determining the elemental composition of unmodified and modified nanoparticles, an XPS model K-ALPHA (Thermo Scientific, Waltham, MA, USA) was used with a monochromatic aluminum (Al Kα) bonding range from 0 to 1350 eV, with a depth of 400 μm.

Finally, the ASTM standard E2149-01 [31] was used to determine the antimicrobial properties of modified and unmodified nanoparticles: Standard Test Method for Determining the Antimicrobial Activity of Immobilized Antimicrobial Agents Under Dynamic Contact Conditions.

The strains used in this study were *Escherichia coli* and *Staphylococcus aureus* (Gram-negative and Gram-positive bacteria, respectively).

The preparation of the inoculum consisted of the propagation of the strains of *Escherichia coli* and *Staphylococcus aureus*, which was carried out by using a liquid culture in trypticase soy broth for 24 h at a temperature of 37 °C on a New Brunswick Scientific mechanical shaker at 180 rpm. The recovery of the biomass of each of the cultures of each strain of the microorganism to be evaluated was carried out by centrifuging the suspension contained in the flask at 14,000 rpm, at a temperature of 20 °C for 15 min, and three washes of the biomass were carried out with a physiological solution (0.85% NaCl).

The biomass recovered from each microorganism was resuspended in 20 mL of physiological solution to subsequently determine the number of colony-forming units per milliliter of suspension (CFU/mL) using a viable count of each suspension. Under sterile conditions, the viable count was performed by taking 1 mL of the cell suspension and adding it to a test tube containing 9 mL of physiological solution. The tube was covered and shaken vigorously to take 1 mL of it and dilute it again in another tube with the same volume of physiological solution. Another milliliter of the solution contained in the tube was taken again and placed in a sterile Petri dish with 20 mL of trypticase soy agar. This procedure was repeated until the sample was diluted six times. The dishes were incubated at 37 °C for 24 h in a Lab. Line Instruments incubator. At the end of the incubation time, the plates were removed from the incubator, and the CFU/mL was counted to determine the number of bacteria present in the cell suspension. This procedure was repeated for each of the cell suspensions of each of the microorganism strains used in the evaluation. Based on the number of bacteria present in the cell suspension, convenient calculations were made to adjust the concentration of the inoculum to the value specified by the standard (20,000 CFU/mL) with trypticase soy broth diluted 1/500.

For the inoculation of the samples and the initiation of the antimicrobial activity test of the modified and unmodified nanoparticles, sterile vials with a capacity of 5 mL were used. The target, in this case, was the inoculation of the microorganism to be evaluated without the addition of nanoparticles. Under aseptic conditions, the modified nanoparticles were placed according to the different concentrations used (200, 500, 800, 1100, and 1400 ppm) in the vials, where 5 mL of 1/500 soy broth was added and inoculated with approximately 100 μL of the suspension of the microorganisms to be evaluated, then the vials were covered with cotton plugs. Assigned samples were placed in a Precision Scientific incubator at 37 °C and 90% humidity for 24 h. At the end of the incubation period, the samples were removed from the incubator, and the bacterial count was performed as described above. The Petri dishes were incubated at 37 °C for 24 h. Once the incubation time was over, the bacterial count was carried out within 24 h of the nanoparticles being exposed to the different microorganisms.

## 3. Results and Discussion

### 3.1. Nanoparticle Synthesis

The surface modification was carried out using the ultrasound method, using LA and SA as organic binders. The surface modification was analyzed using TGA, DSC, XRD, FTIR, and XPS techniques. The results obtained are presented and discussed below. To facilitate comprehension, all samples have been systematically labeled. Table 1 shows the modification treatments, reaction times, and organic acid used for each of the nanoparticles.

#### 3.1.1. Thermogravimetric Analysis (TGA)

The TGA analysis of the TiO_2_ and ZrO_2_ nanoparticles’ surface modified with the organic acids presented information on the content of the organic binder. The quantity of binder bound on the surface of the nanoparticles is contingent upon the type of carboxylic acid utilized and the reaction time employed during the modification process.

**Figure 1:**
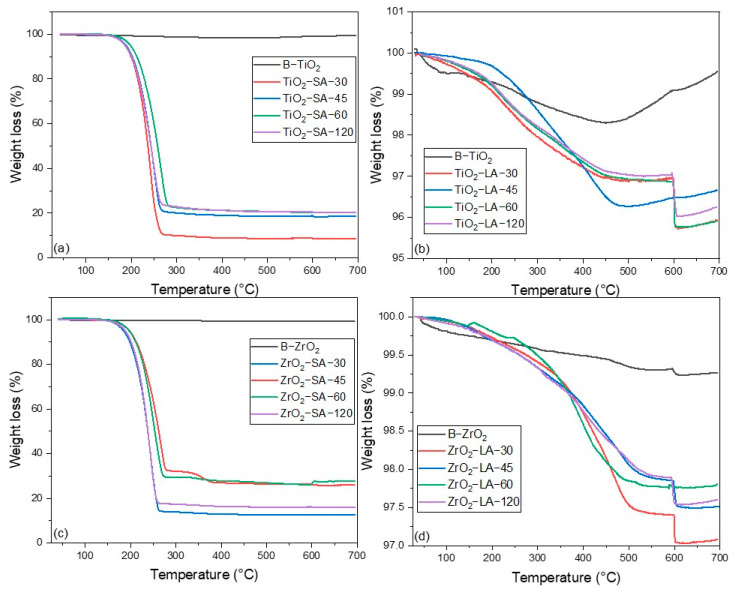
TGA of unmodified and modified TiO_2_ and ZrO_2_ nanoparticles. (**a**) SA-modified TiO_2_ nanoparticles, (**b**) LA-modified TiO_2_ nanoparticles, (**c**) SA-modified ZrO_2_ nanoparticles, (**d**) LA-modified ZrO_2_ nanoparticles.

The thermograms of the modified and unmodified B-TiO_2_ and B-ZrO_2_ nanoparticles are presented in Figure 1. In the temperature range below 200 °C, a mass loss of less than 0.5% is observed in both samples. This phenomenon can be attributed to the presence of volatile compounds during the synthesis of nanoparticles or traces of moisture (Table 2).

The nanoparticles modified with SA had a higher organic binder content compared to those modified with LA. Table 2 presents the data obtained in the TGA analysis; the inorganic residue (IR) corresponds to the metal oxide, while the organic binder content (OB) belongs to the amount of SA or LA.

The thermograms of the modified nanoparticles show graphs with different characteristics; for the nanoparticles modified with SA (Figure 1a,c), there is a mass loss that begins at 200 °C and ends at 300 °C. The thermogram of the LA-modified nanoparticles (Figure 1b,d) exhibits continuous mass loss in the range of 150 °C to 500 °C.

The ZrO_2_ nanoparticles modified with SA (Figure 1c) at an ultrasound process time of 30 min present the highest % of OB (86.4%). As the ultrasound process time increases, the % of OB decreases, but at a time of 120 min, an increase in the % is observed from OB; this could be attributed to the fact that with a longer sonication time, the nanoparticles could fragment, and subsequently, the fragments could be modified by increasing the % OB. For ZrO_2_ nanoparticles modified with LA (Figure 1d), a trend is shown to decrease the % OB as the sonication treatment time increases, although the percentages of OB are lower with LA than with SA.

In the case of TiO_2_ nanoparticles, they exhibit different behaviors. TiO_2_ nanoparticles modified with SA (Figure 1a) show a trend concerning the ultrasound processing time; as this time increases, the % OB decreases. On the other hand, for TiO_2_ nanoparticles modified with LA (Figure 1b), there is no trend since they show increases and decreases in % OB as the ultrasound processing time. The highest %OB for TiO_2_ nanoparticles with SA and LA is 90.3% and 3.25%, respectively.

The thermograms are depicted in Figure 1, and the data are presented in Table 2. The following dataset contains the TGA of unmodified and modified TiO_2_ and ZrO_2_ nanoparticles. We demonstrate that the surface modification with SA was more efficient than that with LA. The percentage of the SA binder on the surface of ZrO_2_ nanoparticles is between 72.0 and 86.4%, while for the LA binder, it is 1.23–2.45%. On the other hand, the percentage of binder present in the TiO_2_ nanoparticles ranged from 78.4 to 90.3% for SA and from 2.75 to 3.25% for the LA binder. The difference in results can be attributed to the chemical structure of the organic binders and their solubility. Wenhui Li et al. made polylactic acid composite films containing different percentages (*w*/*w*) of TiO_2_ nanoparticles, reporting a polylactic acid degradation temperature close to 286 °C [32]. On the other hand, steric acid degradation temperatures close to 217 °C have been reported, as shown by Shengli Niu et al. [33].

#### 3.1.2. Differential Scanning Calorimetry Analysis (DSC)

**Figure 2:**
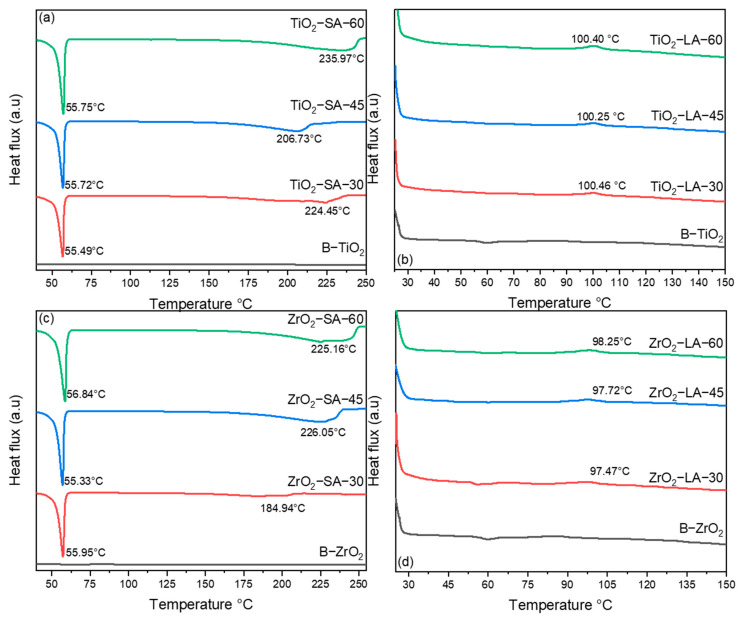
DSC curves for TiO_2_ and ZrO_2_ unmodified and modified nanoparticles. (**a**) TiO_2_-SA nanoparticles, (**b**) TiO_2_-LA nanoparticles, (**c**) ZrO_2_-SA nanoparticles, (**d**) ZrO_2_-LA nanoparticles.

Figure 2 shows the DSC thermograms obtained from the TiO_2_ and ZrO_2_ nanoparticles modified with SA and LA. The thermograms corresponding to the unmodified nanoparticles (B-TiO_2_ and B-ZrO_2_) are also presented for comparative purposes.

As illustrated in Figure 2a, the TiO_2_ nanoparticles modified with SA exhibit an exothermic transition at approximately 55 °C, which is attributed to the melting temperature (Tm) of the SA binder. The Tm reported for SA is 67.7 °C [34]; the lower value obtained in the present investigation is attributed to the fact that the SA binder within the functionalization process can form nanometric-sized particles. The calorimetric curves present an increase in heat flux at temperatures close to 240 °C, which may be indicative of a degradation process of the SA binder; a similar situation was previously described [35].

As illustrated in Figure 2b, the thermograms of the TiO_2_ nanoparticles modified with LA reveal the presence of these materials. The calorimetric curves yielded minimal information regarding the LA binder. At approximately 100 °C, an endothermic transition was observed, which may be indicative of water evaporation and possible condensation of the binder.

The DSC thermograms belonging to the ZrO_2_ nanoparticles modified with SA and LA are presented in Figure 2c,d, and similarly, the same situation was observed in the thermograms of the TiO_2_ nanoparticles modified with SA and LA.

#### 3.1.3. Wide-Angle X-Ray Diffraction (WAXD) Analysis

**Figure 3:**
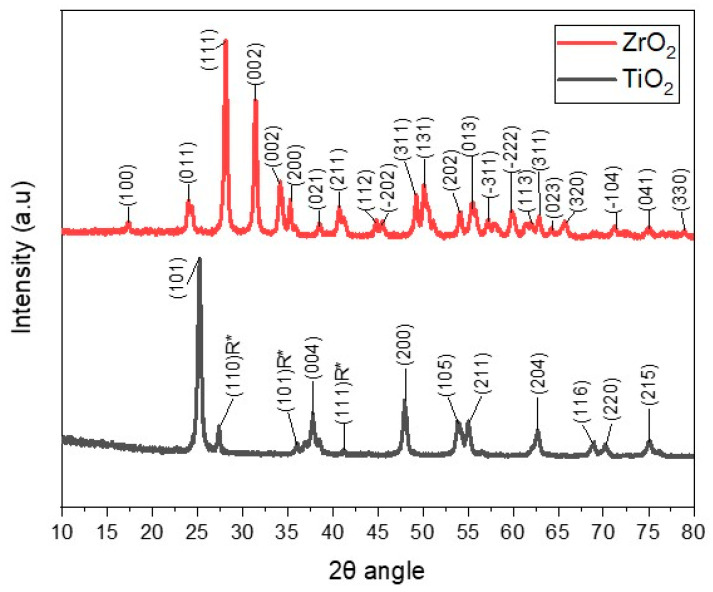
X-ray diffraction analysis of ZrO_2_ and TiO_2_ nanoparticles.

The commercial TiO_2_ and ZrO_2_ nanoparticles were characterized by X-ray diffraction (Figure 3). The XRD pattern of ZrO_2_ nanoparticles recorded in the range 2θ = 10° to 80° presented several peaks, located at 24.07°, 28.19°, 31.49°, 34.21°, 35.31°, 38.56°, 40.73° and 41.25° at angles 2θ [36]. The diffractogram of the TiO_2_ nanoparticles exhibits diffraction signals corresponding to titanium oxide in its anatase phase, which are found at 25.27°, 37.68°, 47.99°, 53.79°, 55.07°, 62.72°,68.93°, 70.27°, and 75.05° at angles 2θ [37]. Some rutile phase (R*) diffraction peaks of TiO_2_ with very low intensities were also identified [38].

The commercial TiO_2_ and ZrO_2_ nanoparticles had an average crystallite size (D) of 16.60 nm and 19.65 nm, respectively, which were determined using the Debye–Scherrer formula, considering the diffraction peak with the highest intensity located at 2θ of 25.27° and 28.1 ± 19° for TiO_2_ and ZrO_2_, respectively.

**Figure 4:**
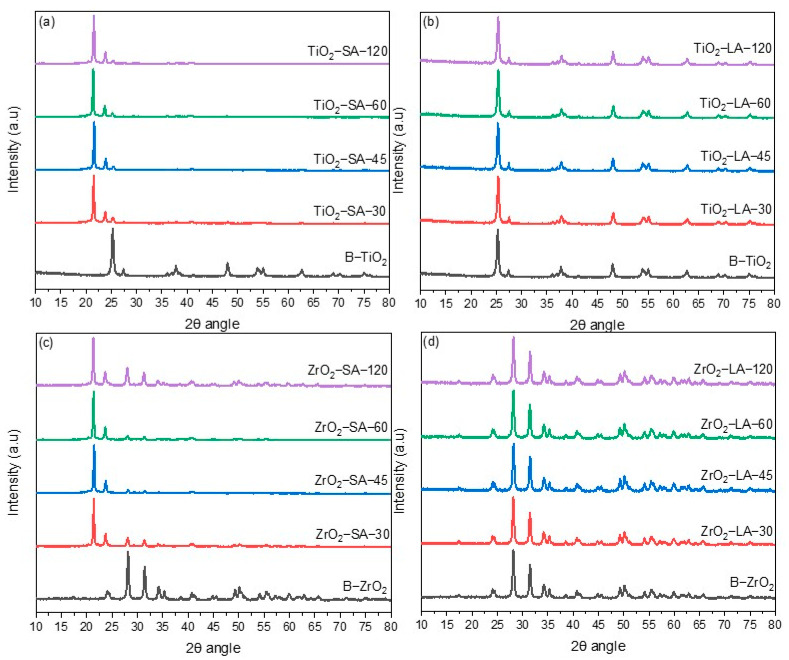
XRD analysis of TiO_2_ and ZrO_2_ unmodified and modified nanoparticles. (**a**) SA-modified TiO_2_; (**b**) LA-modified TiO_2_; (**c**) SA-modified ZrO_2_; (**d**) LA-modified ZrO_2_.

Furthermore, XRD also analyzed the modified and unmodified TiO_2_ and ZrO_2_ nanoparticles. Figure 4 shows the diffractograms obtained from the modified nanoparticles of TiO_2_ (Figure 4a,b) and ZrO_2_ (Figure 4c,d) at different reaction times, which are presented alongside the diffractograms corresponding to the unmodified nanoparticles.

The diffractograms of the TiO_2_ and ZrO_2_ nanoparticles superficially modified with LA (Figure 4b,d) show a similar pattern to the unmodified particles. Furthermore, no shifts are observed in the diffraction peaks of the TiO_2_ and ZrO_2_ nanoparticles; this suggests that the nanoparticles’ crystallinity is not affected by the interaction with LA, which can be attributed to the low % of OB, as shown in the previous TGA analysis.

For the SA-modified nanoparticles (Figure 4a,c), the peaks corresponding to metal oxides are shown, and two other peaks located at 2θ angles of 21.44° and 23.76° are associated with the presence of SA [39]. This phenomenon can be attributed to the elevated organic binder (SA) content observed in these samples, which surpasses that of the LA-containing nanoparticles. This information shows a greater interaction between the SA and the TiO_2_ and ZrO_2_ nanoparticles.

The XRD technique was employed to ascertain the mean crystallite size (D) of the modified nanoparticles. This was accomplished by calculating the mean crystallite size using the Debye–Scherrer equation, with the highest intensity peak being taken into consideration. Table 3 shows the calculated results. LA surface modification of TiO_2_ and ZrO_2_ nanoparticles causes slight changes in the D. In the ZrO_2_ nanoparticles, the size decreased slightly compared to the unmodified sample; only in the ZrO_2_-LA-30 sample was an increase in size observed. The behavior was different for the TiO_2_ nanoparticles. The size increased slightly compared to the original sample. The TiO_2_-LA-45 sample presented the highest increase (0.55 nm). During the ultrasound processing, the size of the nanoparticles initially decreases due to the dispersion energy; if the particles do not stabilize, an increase in size may occur [40].

In TiO_2_ and ZrO_2_ nanoparticles modified with SA, there was a decrease in D concerning the unmodified nanoparticles; in general, it was observed that the size decreases as the ultrasound processing time increases. The largest decrease was 2.29 and 4.8 nm and corresponded to the ZrO_2_-SA-120 and TiO_2_-SA-120 samples, respectively. The decrease in the average crystallite size could have an important effect on the antibacterial activity. It has been reported that nanoparticles with small crystal sizes show greater antibacterial activity compared to those with larger crystal sizes [41,42,43,44].

#### 3.1.4. Fourier Transform InfraRed (FTIR) Analysis

**Figure 5:**
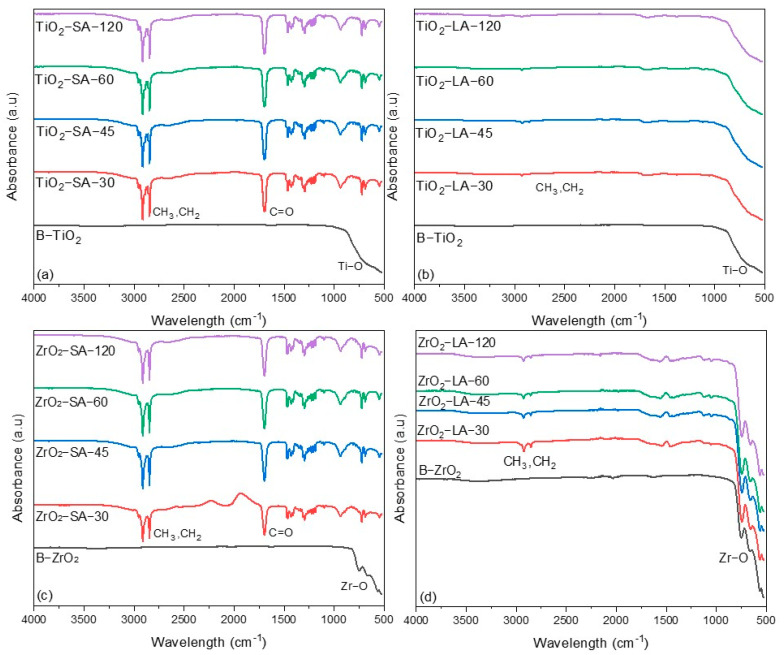
FTIR spectra of unmodified and modified TiO_2_ and ZrO_2_ nanoparticles: (**a**) SA-modified TiO_2_; (**b**) LA-modified TiO_2_; (**c**) SA-modified ZrO_2_; (**d**) LA-modified ZrO_2_.

FTIR spectroscopy of the modified and unmodified TiO_2_ and ZrO_2_ nanoparticles was carried out to detect the presence of organic binders and metal oxides (Figure 5). For comparison purposes, the FTIR spectrum of the unmodified nanoparticles is located at the bottom of these spectra.

The nanoparticles modified with SA presented the characteristic signals of SA (Figure 5a,c). The large amount in which this compound is found facilitates its determination, but makes the detection of TiO_2_ and ZrO_2_ difficult. In the case of TiO_2_, the characteristic bands are located at 483 cm^−1^ (Ti-O) and 623.5 cm^−1^ (Ti-OH) [45], while for ZrO_2,_ a vibration band of the Zr-O bond is reported between 500 and 850 cm^−1^ [46], the presence of the oxides is not ruled out by FTIR spectroscopy because the expected bands may fall under the intense signals of the SA binder.

The FTIR characterization of the LA-modified TiO_2_ and ZrO_2_ nanoparticles (Figure 5b,d) showed several broad bands in the range of 500–850 cm^−1^. This set of bands could indicate the presence of the oxides of interest. In the samples modified with LA, the signals of the functional groups of the organic binder, such as C = O and -OH, cannot be seen; due to the low content of this binder, for this reason, its detection using FTIR spectroscopy is difficult. The spectra obtained only presented very weak bands in the range of 2700–2950 cm^−1^ that indicate the presence of aliphatic chains in the sample, that is, the presence of CH_3_ and CH_2_ groups. The LA signals have been referenced at 2994.5 cm^−1^ and 2977.9 cm^−1^, corresponding to vibrations of asymmetric and symmetric CH_2_ groups, respectively [47]. Characteristic signals of SA have also been reported at 1705 cm^−1^ (C = O) and between 2700 cm^−1^ and 3000 cm^−1^, corresponding to vibrations of CH_3_ and CH_2_ groups [48].

#### 3.1.5. X-Ray Photoelectron Spectroscopy (XPS) Analysis

**Figure 6:**
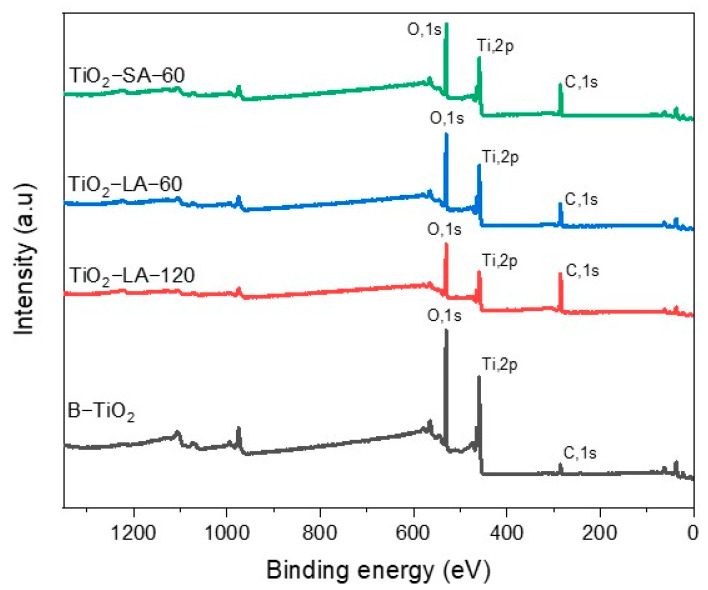
XPS analysis of unmodified and modified TiO_2_ nanoparticles.

Figure 6 shows the results of the XPS analysis for the TiO_2_ nanoparticle with and without surface modification. In the case of modified TiO_2_, characteristic photoemitted electron signals were observed at 458.8 eV for Ti2p, 530.22 eV for O1s [49], and the presence of a carbon signal at 284.91 eV, confirming the presence of organic ligands. On the other hand, unmodified B-TiO_2_ nanoparticles presented binding energy signals at 458.84 eV and 530.31 eV for Ti2p and O1s, respectively. It is also observed the presence of a carbon signal at 284.9 ± 0.2 eV. This is due to the sample preparation, since carbon or copper tapes are used very frequently to support the samples and be analyzed by XPS. Therefore, considering that the detection sensitivity of the equipment is 0.1%, the C1 element was detected. For the analysis of the atomic propensity of the surface-modified nanoparticles, the signal of the photoemitted electrons C1s was considered null, such that it did not influence the percentages of the other elements. The corresponding results are shown in Table 4. Binding energies and atomic percentages of TiO_2_ nanoparticles.

Table 4 presents the results obtained from XPS analysis of TiO_2_ nanoparticles with and without surface modification. It shows the binding energies of each element and its atomic composition.

**Figure 7:**
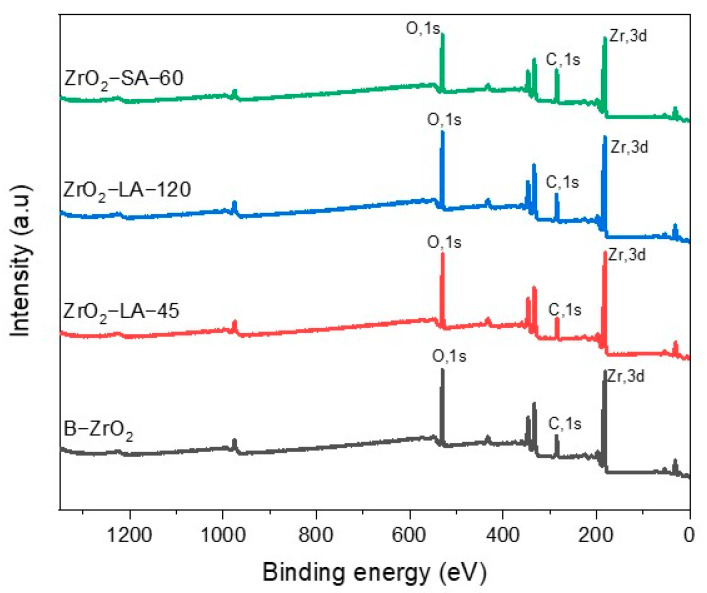
XPS analysis of unmodified and modified ZrO_2_ nanoparticles.

Figure 7 shows the XPS spectra of the modified and unmodified ZrO_2_ nanoparticles. For the B-ZrO_2_ nanoparticle (located at the bottom), characteristic signals of photoemitted electrons of O1s at 530.19 eV and of Zr 3d at 182.08 eV are observed, which agrees with what is reported in the literature [40]. Also, it presents the signal of C1s due to the above-mentioned. For the modified ZrO_2_ nanoparticles, similar values are shown in the photoemitted electron signals for O1s at 530.08 eV, Zr3d at 182.08 eV, and a slight increase in the intensity of the C1s signal at 284.95 eV.

In general terms, Table 5 shows the atomic% of the modified and unmodified nanoparticles; the sample that has a higher % of C1s is ZrO_2_-SA with 48.74%, which corroborates the presence of organic matter on the surface of the ZrO_2_ nanoparticles modified with the different organic acids.

Table 5 presents the data from the XPS analysis in terms of binding energies and atomic percentage of the modified and unmodified ZrO_2_ nanoparticles.

In general, in the modified ZrO_2_ nanoparticles (with SA and LA), there are no considerable shifts in the characteristic signals of the photoemitted electrons for C1s, O1s, and Zr3d. The atomic percentage of the modified nanoparticles shows some significant variations related to the type of organic binder and the ultrasonic treatment time. For example, the sample with the lowest percentage of C1s was TiO_2_-LA-45, with 33.28%. In this case, it is suggested that the shorter the ultrasound treatment time, the lower the % of C1s will be. The TiO_2_ nanoparticles modified with different organic acids show similar results to those described above since they do not show significant shifts in the signals of the photoemitted electrons of C1s, O1s, and Ti2p. Changes are observed in the atomic % of C1s in the modified nanoparticles; the sample that has the highest % of C1s is TiO_2_-LA-120, with 59.21%. For the TiO_2_ nanoparticles modified with LA, it is suggested that a longer ultrasonication time will be the higher the % of C1s; in turn, this increase could be attributed to a greater interaction of the LA with the surface of the TiO_2_ nanoparticles. Something similar has already been described, where the % of C1s increased over the time of ultrasonic processing [50].

#### 3.1.6. Deconvolution O1s NPs X-Ray Photoelectron Spectroscopy (XPS) Analysis

**Figure 8:**
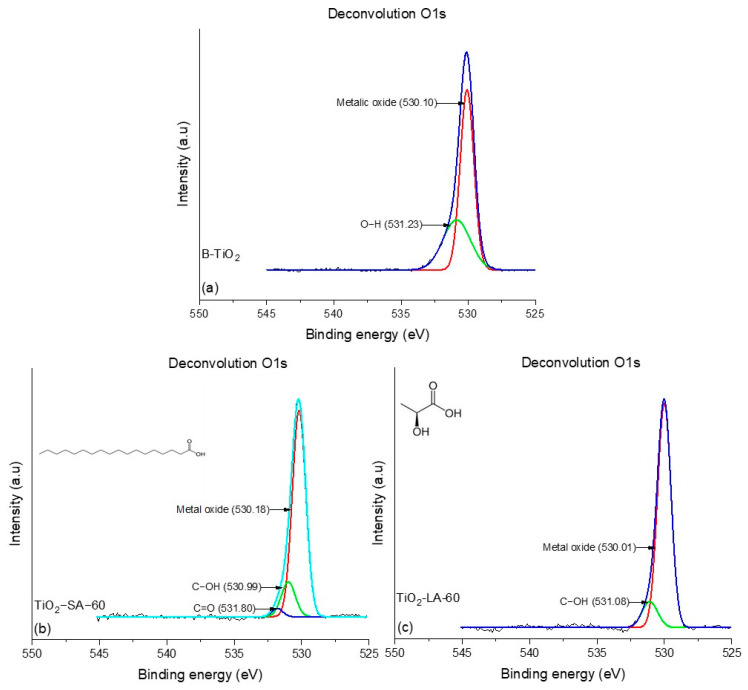
Deconvolution for O1s of samples: (**a**) B-TiO_2_, (**b**) TiO_2_-SA-60, and (**c**) TiO_2_-LA-60.

To analyze the metal oxides and the chemical environment of oxygen atoms and their possible coordination with other elements when carrying out the surface modification of nanoparticles, the deconvolution of the O1s signal of modified NPs for 60 min, and the unmodified NPs was performed. Figure 8 shows the deconvolution of the O1s peaks of TiO_2_ nanoparticles modified with LA and SA and unmodified. Oxygen deconvolution for the B-TiO_2_ nanoparticles (Figure 8a) showed two signals, 530.10 eV and 531.23 eV, the first one related to the H-O bonds of the water molecules adsorbed on the surface of the NPs, and the second due to the metal oxide of NPs (Ti-O), as reported by the literature [51,52]. In the TiO_2_-SA-60 sample (Figure 8c), three signals were observed corresponding to the C=O, C-OH, and metal oxide at 531.80, 530.99, and 530.18 eV, respectively, while TiO_2_-LA-60 nanoparticles present two signals corresponding to C-OH and metal oxide with binding energy values of 531.01 and 530 eV, respectively. This indicates that SA could form a greater number of double bonds, C = O, on the surface of the nanoparticle with the help of continuous frequency ultrasonic energy; this is consistent with what was presented in the FTIR section.

**Figure 9:**
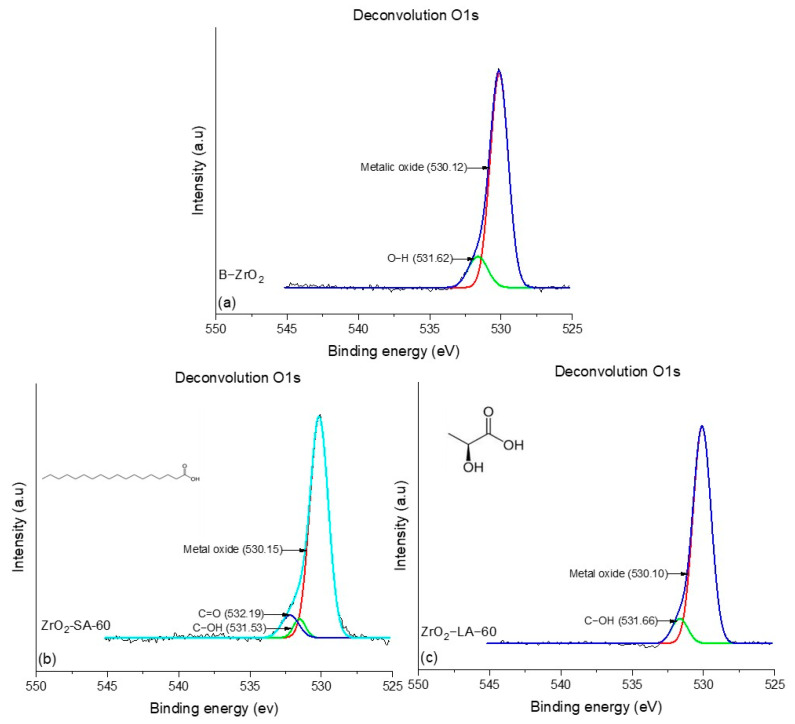
Deconvolution for the O1s of samples (**a**) B-ZrO_2_, (**b**) ZrO_2_-SA-60, and (**c**) ZrO_2_-LA-60.

The deconvolutions of modified and unmodified NPs of ZrO_2_ are presented in Figure 9. The unmodified NPs (B-ZrO_2_) (Figure 9a) presented two signals at 530.12 and 531.62 eV, corresponding to metal oxide (Zr-O) and O-H bonds. Similarly, the ZrO_2_-LA-60 nanoparticles (Figure 9c) showed signals attributed to metal oxide bonds at 530.10 eV and C-OH at 531.40 eV. In contrast, the NPs ZrO_2_-SA-60 (Figure 9b) presented three signals at 530.15, 531.53, and 532.19 eV, corresponding to Zr-O, C-OH, and C = O, as reported by the literature [53]. In general, the analysis of deconvolution of the O1s signal shows the differences between the modified nanoparticles with different organic ligands, as well as shows that both SA-modified nanoparticles have a higher presence of binding interactions between the nanoparticles and their functional groups. This can be attributed to differences in the chemical nature of the organic acids used for surface modification. The results of the deconvolution analysis are in agreement with those obtained by FTIR since the functional groups of the SA were easily detectable in the equipment due to the high content of the same on the surface of the NPs.

### 3.2. Antimicrobial Test

The antibacterial activity (AA) of the nanoparticles was carried out against *Escherichia coli* and *Staphylococcus aureus* (Gram-positive and Gram-negative bacteria, respectively), based on ASTM E2149-01 standard: Standard Test Method for Determining the Antimicrobial Activity of Immobilized Antimicrobial Agents Under Dynamic Conditions. In the study of antimicrobial activity, the minimum bactericidal concentration was determined according to the standard.

**Figure 10:**
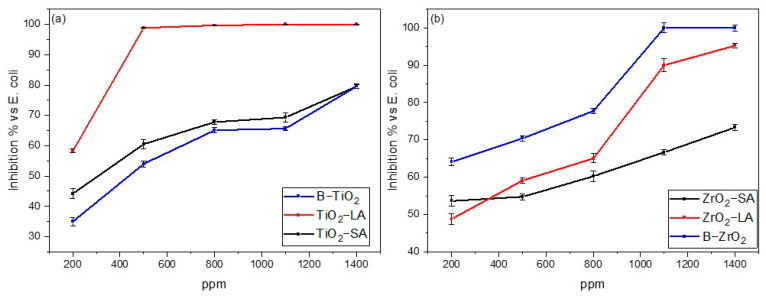
Antimicrobial activity against *Escherichia coli* of: (**a**) modified and unmodified TiO_2_ nanoparticles; and (**b**) modified and unmodified ZrO_2_ nanoparticles.

**Figure 11:**
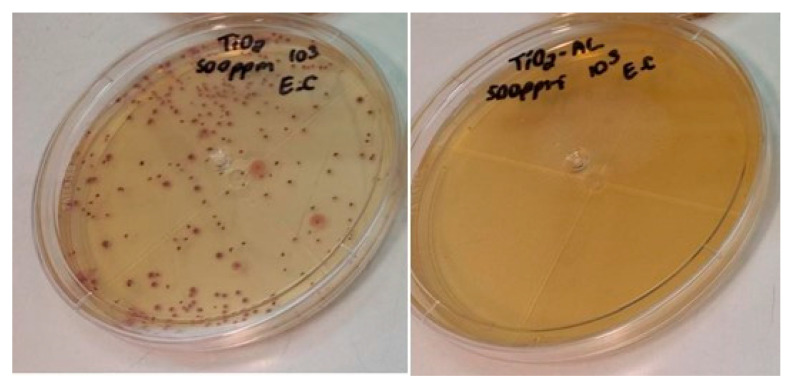
Antimicrobial activity B-TiO_2_ (left) and TiO_2_-LA (right) (500 ppm) against *Escherichia coli*.

The results of the study of the antibacterial activity of the modified and unmodified nanoparticles are presented below. It should be noted that nanoparticles with a reaction time of 60 min were selected to evaluate the performance of antimicrobial activity. This is due to the important results obtained using the techniques described above. In Figure 10 and Figure 11, the results obtained from the percentage of growth inhibition of the different bacteria using modified and unmodified TiO_2_ and ZrO_2_ nanoparticles with both organic ligands were graphed for comparative purposes. The nanoparticle concentrations used were 200, 500, 800, 1100, and 1400 ppm. The contact time for both bacteria was 24 h. In Figure 10 a,b, the results of the antibacterial activity for the *Escherichia coli* bacteria are presented. In general, it is observed that the inhibition of bacterial growth increases as the concentration of nanoparticles increases. The results of the study indicate that the modified nanoparticles exhibit a biocidal effect. Furthermore, these results are consistent with those of most antimicrobial activity studies conducted using various types of nanoparticles. [54,55,56,57,58,59].

The graphs in Figure 10a,b show that B-ZrO_2_ nanoparticles presented greater activity than the B-TiO_2_ nanoparticles against *Escherichia coli* bacteria. The modification of both nanoparticles with SA and LA produced a different behavior; in the specific case of modified TiO_2_ nanoparticles, an increase in antimicrobial activity was observed, and the antimicrobial effect was greater in the nanoparticles modified with lactic acid (TiO_2_-LA). The effect of the modification on the ZrO_2_ nanoparticles was different, as a decrease in the antimicrobial activity was observed. The decrease was more evident in those nanoparticles modified with stearic acid (ZrO_2_-SA), which practically presented the lowest antimicrobial activity at concentrations of 500, 800, 1100, and 1400 ppm. In summary, the modification with SA did not enhance the antimicrobial activity in the ZrO_2_ nanoparticles; however, the TiO_2_ nanoparticles exhibited slightly enhanced antimicrobial activity. Surface modification of TiO_2_ with the LA binder increased the antimicrobial activity against *Escherichia coli* bacteria, achieving 99.0% inhibition at a concentration of 500 ppm.

According to literature data, nanoparticles that can inhibit more than 80% can be considered antimicrobial, among which we have nanoparticles such as TiO_2_-LA and ZrO_2_-LA. The increase in antimicrobial activity of the TiO_2_ nanoparticles modified with LA suggests that the functionality of LA (carboxylic acid and hydroxyl) could increase the formation of reactive oxygen species (ROS), producing harmful effects on the cell walls of bacteria, which generates a state of oxidative stress in them, leading to their death.

Modification with SA does not represent a good alternative to increase the antimicrobial activity of the nanoparticles. This is because this organic binder has in its chemical structure an aliphatic chain that covers the surface of the nanoparticles and avoids chemical interactions with water; consequently, the release of metal ions necessary for antimicrobial activity is not favored. Despite the fact that the SA-modified NPs demonstrated low efficiency in eradicating bacteria, they have the potential to be utilized in conjunction with resins to form hydrophobic coatings, as has been observed in other studies [19]. Figure 11 shows a photograph of *Escherichia coli* bacteria colonies after being treated with 500 ppm of TiO_2_ NPs without and modified with LA.

**Figure 12:**
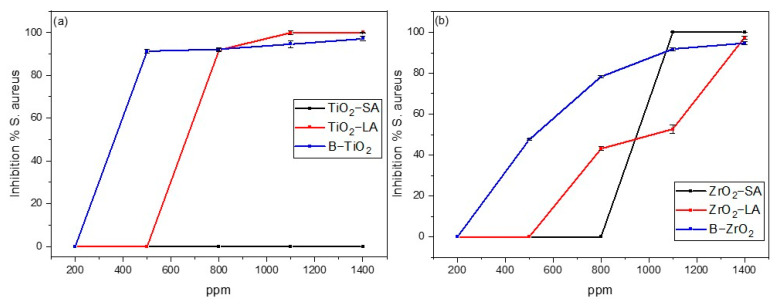
Antimicrobial activity against *Staphylococcus aureus* of: (**a**) modified and unmodified TiO_2_ nanoparticles; and (**b**) modified and unmodified ZrO_2_ nanoparticles.

The antimicrobial study of the TiO_2_ and ZrO_2_ nanoparticles was also carried out for *Staphylococcus aureus* with an exposure time of 24 h. Figure 12a,b shows the percentage of bacterial inhibition versus nanoparticle concentration graphs.

As demonstrated in the graphs of Figure 12, the B-TiO_2_ and B-ZrO_2_ NPs exhibited remarkable antimicrobial activity against the bacterium *Staphylococcus aureus.* The B-TiO_2_ nanoparticles were more efficient, achieving bacterial inhibition greater than 80% at concentrations of 500 ppm, while B-ZrO_2_ requires 800 ppm.

**Figure 13:**
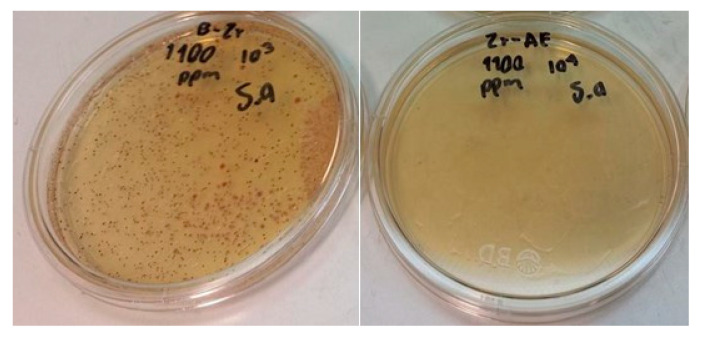
Antimicrobial activity B-ZrO_2_ (left) and ZrO_2_-SA (right) (1100 ppm) against *Staphylococcus aureus*.

Surface modification of B-TiO_2_ and B-ZrO_2_ NPs with SA and LA produced different results than those observed in AA against coli bacteria, TiO_2_-LA, ZrO_2_-SA, and ZrO_2_-LA NPs reached 80% inhibition at concentrations of 800 ppm, 1200 ppm, and 1400 ppm respectively. The AA was very similar to that of unmodified nanoparticles (B-TiO_2_ and B-ZrO_2_). In the case of TiO_2_-SA NPs, the modification with SA produced a negative effect on AA at all concentrations studied. Figure 13 shows a photograph of *Staphylococcus aureus* bacteria colonies after being treated with 1100 ppm of ZrO_2_ NPs without and modified with SA.

The surface modification of both NPs had no significant effects on AA; at concentrations below 600 ppm, it had negative effects. No definitive conclusion can be drawn on the potential impact of NP surface modification on its efficacy against *Staphylococcus aureus* bacteria. However, this could be a crucial consideration in applications where NPs must exhibit easy dispersibility, such as in the context of antimicrobial coatings. Nanoparticles considered to be antibacterial against *Staphylococcus aureus* were TiO_2_-LA, ZrO_2_-SA, and ZrO_2_-LA.

As noted above, surface modification of B-TiO_2_ and B-ZrO_2_ NPs with SA leads to a decrease in crystallite size, although it is logical to assume that AA should be better at smaller crystal sizes; the results presented demonstrate the opposite (with some exceptions), suggesting that the nature of the ligand plays a more important role in AA modification, LA ligand which presents two functionalities in its chemical structure (COOH and ROH) had a greater impact on AA.

According to research by Toledo et al., the AA analysis of ZnO nanoparticles and citric acid functionalized ZnO nanoparticles with crystal sizes of 30.1 nm and 47.9 nm, respectively, yielded similar results. Unexpectedly, the larger NPs exhibited higher AA against *Escherichia coli* and *Staphylococcus aureus* bacteria [60].

## 4. Conclusions

The surface modification of the TiO_2_ and ZrO_2_ nanoparticles with the organic binders SA and LA was successfully achieved using continuous frequency ultrasound with a catenoidal probe at a wave amplitude of 50% and with an energy of 750 W. The analysis by different characterization techniques showed that the synthesized materials contain metal oxides and organic coatings.

XRD analysis revealed the crystalline identity of the employed nanoparticles, anatase for TiO_2,_ and a cubic structure for ZrO_2_ nanoparticles. TGA showed the percentage of organic binders present on the surfaces of the nanoparticles modified with SA and LA, and the presence of organic binders on the surface of the nanoparticles was confirmed using FTIR and XPS.

Finally, it is concluded that, although surface modification with ultrasound and organic ligands produces smaller nanoparticles, their effect on increasing AA against *Escherichia coli* and *Staphylococcus aureus* bacteria is moderate. The elevated AA observed in LA-modified NPs suggests that more polar ligands enhance the AA properties.

Functionalized NPs, which increase their AA, could have great advantages over non-functionalized NPs because they can be more easily dispersed in different media, such as paints, polymers, etc. The employment of SA-functionalized NPs in the fabrication of hydrophobic coatings has the potential to serve as a strategy for achieving the elimination of bacteria through physical mechanisms.

## Figures and Tables

**Table 1:** Modification treatments of TiO_2_ and ZnO nanoparticles.

Sample	Surface Modifier	Reaction Time (min)	Sample	Surface Modifier	Reaction Time (min)
B-TiO_2_	-	-	B-ZrO_2_	-	-
TiO_2_-SA-30	SA	30	ZrO_2_-SA-30	SA	30
TiO_2_-SA-45	SA	45	ZrO_2_-SA-45	SA	45
TiO_2_-SA-60	SA	60	ZrO_2_-SA-60	SA	60
TiO_2_-SA-120	SA	120	ZrO_2_-SA-120	SA	120
TiO_2_-LA-30	LA	30	ZrO_2_-LA-30	LA	30
TiO_2_-LA-45	LA	45	ZrO_2_-LA-45	LA	45
TiO_2_-LA-60	LA	60	ZrO_2_-LA-60	LA	60
TiO_2_-LA-120	LA	120	ZrO_2_-LA-120	LA	120

**Table 2:** TGA data of unmodified and modified TiO_2_ and ZrO_2_ nanoparticles.

Sample	IR%; (OB%)	Sample	IR%; (OB%)
B-TiO_2_	99.0	B-ZrO_2_	99.5
TiO_2_-SA-30	8.72; (90.3)	ZrO_2_-SA-30	12.66; (86.4)
TiO_2_-SA-45	18.62; (80.4)	ZrO_2_-SA-45	25.95; (73.6)
TiO_2_-SA-60	20.47; (78.5)	ZrO_2_-SA-60	27.61; (72.0)
TiO_2_-SA-120	20.61; (78.4)	ZrO_2_-SA-120	16.04; (83.5)
TiO_2_-LA-30	95.75; (3.25)	ZrO_2_-LA-30	97.06; (2.44)
TiO_2_-LA-45	96.25; (2.75)	ZrO_2_-LA-45	97.05; (2.45)
TiO_2_-LA-60	95.76; (3.24)	ZrO_2_-LA-60	97.77; (1.23)
TiO_2_-LA-120	96.03; (2.97)	ZrO_2_-LA-120	97.57; (1.43)

**Table 3:** XRD obtained data from TiO_2_ and ZrO_2_ unmodified and modified nanoparticles.

Sample	2θ Angle *; Plane (101)	Crystal Size (nm)	Standard Error (size)	Sample	2θ Angle **; Plane (111)	Crystal Size (nm)	Standard Error (size)
B-TiO_2_	25.25	16.60	0.00234	B-ZrO_2_	28.16	19.65	0.0023
TiO_2_-LA-30	25.34	17.06	0.00225	ZrO_2_-LA-30	28.14	19.88	0.00247
TiO_2_-LA-45	25.31	17.15	0.00241	ZrO_2_-LA-45	28.18	19.30	0.0029
TiO_2_-LA-60	25.34	17.10	0.00229	ZrO_2_-LA-60	28.14	19.21	0.00268
TiO_2_-LA-120	25.33	16.98	0.00242	ZrO_2_-LA-120	28.17	19.52	0.00276
TiO_2_-SA-30	25.29	13.21	0.00605	ZrO_2_-SA-30	28.09	17.93	0.00104
TiO_2_-SA-45	25.37	12.30	0.00836	ZrO_2_-SA-45	28.16	17.85	0.00366
TiO_2_-SA-60	25.17	12.39	0.00858	ZrO_2_-SA-60	28.08	17.76	0.00381
TiO_2_-SA-120	25.32	11.76	0.00577	ZrO_2_-SA-120	28.01	17.36	0.00208

* Standard error of the measurements of the 2θ angle belonging to the plane (101) = 0.0567; ** standard error of the measurements of the 2θ angle belonging to the plane (111) = 0.0521.

**Table 4:** Binding energies and atomic percentages of TiO_2_ nanoparticles.

Sample	C1s (eV)	O1s (eV)	Ti2p (eV)	C1s (at %)	O1s (at %)	Ti2p (at %)
B-TiO_2_	NA	530.31	458.84	NA	69.5	30.5
TiO_2_-LA-120	284.91	530.21	458.73	59.21	29.61	11.18
TiO_2_-LA-60	285.03	530.22	458.87	37.29	43.84	18.87
TiO_2_-SA-60	284.97	530.27	458.8	45.25	38.04	16.71

**Table 5:** Binding energies and atomic percentages of ZrO_2_ nanoparticles.

Sample	C1s (eV)	O1s (eV)	Zr3d (eV)	C1s at %	O1s at %	Zr3d at %
B-ZrO_2_	NA	530.19	182.08	NA	61.18	38.82
ZrO_2_-LA-45	284.37	530.08	182.08	33.28	41.08	25.65
ZrO_2_-LA-120	284.95	530.25	183.17	37.59	38.16	24.25
ZrO_2_-SA-60	284.87	530.21	182.08	48.74	30.93	20.33

## Data Availability

The dates are contained within the article.
